# CCN3 is dynamically regulated by treatment and disease state in multiple sclerosis

**DOI:** 10.1186/s12974-020-02025-7

**Published:** 2020-11-22

**Authors:** Michelle Naughton, Jill Moffat, George Eleftheriadis, Nira de la Vega Gallardo, Andrew Young, John Falconer, Kristen Hawkins, Ben Pearson, Bernard Perbal, Andrew Hogan, Paul Moynagh, Sam Loveless, Neil P. Robertson, Bruno Gran, Rachael Kee, Stella Hughes, Gavin McDonnell, Owain Howell, Denise C. Fitzgerald

**Affiliations:** 1 Wellcome-Wolfson Institute for Experimental Medicine, Queen’s University Belfast, 97 Lisburn Road, Belfast, Northern Ireland BT9 7BL UK; 2 Institute of Life Science, Swansea University Medical School, Swansea, Wales, UK; 3 International CCN Society, Nice, France; 4 Institute of Immunology, Department of Biology, National University of Ireland Maynooth, Maynooth, County Kildare Ireland; 5 Department of Neurology, University Hospital of Wales and Division of Psychological Medicine and Clinical Neurosciences, Cardiff University, Cardiff, UK; 6 Clinical Neurology, Division of Clinical Neuroscience, University of Nottingham School of Medicine, Nottingham, UK/Department of Neurology, Nottingham University Hospitals NHS Trust, Nottingham, UK; 7 Belfast Health and Social Care Trust, Belfast, Northern Ireland UK

**Keywords:** Multiple sclerosis, CCN3, Myelin, Plasma, CSF

## Abstract

**Background:**

Multiple sclerosis (MS) is an immune-mediated disease that damages myelin in the central nervous system (CNS). We investigated the profile of CCN3, a known regulator of immune function and a potential mediator of myelin regeneration, in multiple sclerosis in the context of disease state and disease-modifying treatment.

**Methods:**

CCN3 expression was analysed in plasma, immune cells, CSF and brain tissue of MS patient groups and control subjects by ELISA, western blot, qPCR, histology and in situ hybridization.

**Results:**

Plasma CCN3 levels were comparable between collective MS cohorts and controls but were significantly higher in progressive versus relapsing-remitting MS and between patients on interferon-β versus natalizumab. Higher body mass index was associated with higher CCN3 levels in controls as reported previously, but this correlation was absent in MS patients. A significant positive correlation was found between CCN3 levels in matched plasma and CSF of MS patients which was absent in a comparator group of idiopathic intracranial hypertension patients. PBMCs and CD4^+^ T cells significantly upregulated CCN3 mRNA in MS patients versus controls. In the CNS, CCN3 was detected in neurons, astrocytes and blood vessels. Although overall levels of area immunoreactivity were comparable between non-affected, demyelinated and remyelinated tissue, the profile of expression varied dramatically.

**Conclusions:**

This investigation provides the first comprehensive profile of CCN3 expression in MS and provides rationale to determine if CCN3 contributes to neuroimmunological functions in the CNS.

**Supplementary Information:**

The online version contains supplementary material available at 10.1186/s12974-020-02025-7.

## Background

Multiple sclerosis (MS) is an inflammatory, neurodegenerative disease characterised by central demyelinating events disseminated in time and space. Efficient myelin regeneration (remyelination) declines with age and remains an unmet clinical need that has the potential to be neuroprotective and functionally restorative for patients with all types of MS. With a renewed focus on therapies for progressive MS and regenerative strategies that may prevent further disability, new compounds are being identified to enhance the regeneration of myelin by oligodendrocytes.

Although most MS treatments block either peripheral immune cell function or access to the CNS, immune cells are increasingly recognised as key players in modulating tissue regeneration. Indeed, depletion of T cells or specific subsets such as regulatory T cells (Treg) impedes myelin regeneration in vivo [1, 2]. A protein secreted by T cells called cellular communication network factor 3 (CCN3) was found to increase oligodendrocyte differentiation in experimental models in vitro and ex vivo [1]. CCN3 was previously known as nephroblastoma overexpressed or NOV [3]. It is a matricellular protein of the CCN family (CCN1-6) [4]. These proteins are dynamically expressed in development and injury settings, and have cell regulatory functions rather than structural roles [5–7]. CCN proteins comprise 4 domains which have homology to insulin-like growth factor binding proteins (IGFBPs), von Willebrand factor type C repeat (VWC), thrombospondin type I repeat (TSP1) and a carboxy-terminal domain (CT) containing a cystine knot. As such, CCN proteins can modulate the binding affinity or bioavailability of various ligands as well as directly interact with cell surface receptors [8–10]. In the case of CCN3, this includes Notch, integrins α_v_β_3_ and α_5_β_1_, heparin sulphate proteoglycans (HSPGs) and S100A4 calcium binding protein [11–14]. CCN3 has been ascribed multiple signalling roles that can influence proliferation, differentiation and inflammatory processes in a cell-specific and context-dependent manner including in Treg-mediated oligodendrocyte differentiation [15, 16]. CCN3 plays various roles in the immune system. It is essential for normal functioning of haematopoietic stem cells (HSCs) [17, 18], is an adipokine linked to obesity-induced insulin resistance [19, 20] and is a regulator of cytokine expression in both the periphery and CNS [19, 21, 22]. The CCN3 promoter is a direct target of the FoxO1 transcription factor, which is essential for CD4^+^ T cell fitness [23]. Indeed, Treg-specific deletion of FoxO1 results in lethal inflammation due to loss of tolerance [24], and its expression is reduced in CD4^+^ and CD8^+^ T cells in MS [25]. Given the importance of CD8^+^ and CD4^+^ T cells, in MS, it is pertinent to explore whether CCN3 expression is altered in MS.

We and others have reported that CCN3 is extensively expressed in the murine CNS, primarily by neurons in the cerebral cortex, hippocampus, amygdala, suprachiasmatic nuclei, anterior olfactory nuclei and spinal cord grey matter [26–32]. Despite this, much of its functional significance in the CNS is still to be explored. In humans, CCN3 has so far been detected in developing brain tissue [33–35] and cerebrospinal fluid (CSF) [36, 37]. It is reported to negatively regulate granule precursor neuron proliferation, inhibit axonal callosal projections during development and upregulate CXCL1 and CCL2 in astrocytes in murine brain [21, 29, 38]. CCN3 from regulatory T cells (Treg) enhanced oligodendrocyte differentiation in mixed glial cultures and is upregulated in GFAP^+^ cells and oligodendroglial cells of remyelinating lesions, but CCN3 was not essential for (re)myelination as demonstrated in CCN3^−/−^ mice [1, 32]. Further investigation is necessary to determine whether this modulatory protein with distinct expression in CNS and immune compartments has potential therapeutic value in demyelinating diseases such as MS. To this end, we conducted extensive profiling of CCN3 expression in healthy and MS disease states to answer whether CCN3 is dysregulated in MS and determine whether there is rationale for CCN3-mediated regenerative mechanisms as a therapeutic target for neurological disease.

## Methods

### Participants

This study was approved by the Yorkshire & the Humber - Leeds West Research Ethics Committee (15/YH/1071) and the institutional ethics committee of Queen’s University Belfast. Written informed consent was obtained from all participants in the study according to the Declaration of Helsinki. Blood samples were collected with informed consent from healthy volunteers and from patients attending the MS clinic at Belfast City Hospital (Belfast Health and Social Care Trust). Patients diagnosed with MS according to the 2010 McDonald criteria and receiving natalizumab (Nat), interferon-β (IFN) or no treatment for relapsing-remitting MS (RRMS) or diagnosed as secondary or primary progressive (PrMS) were included in the study. Donors were excluded if under 18 years old, pregnant or had other inflammatory comorbidities as determined by questionnaire and/or review of clinical notes.

Matched plasma samples from MS patients in relapse or in remitting phases of disease were provided by Dr. Bruno Gran, University of Nottingham. Matched samples of plasma and CSF collected during routine neurologic workups between 2012 and 2017 from age- and sex-matched cohorts with RRMS or idiopathic intracranial hypertension (IIH) were obtained from the Welsh Neuroscience Research Tissue Bank, Cardiff University (Rec Ref. 14/WA/0073). Patients with obesity (BMI > 30) were recruited from the Weight Management Service at St Columcille Hospital led by Prof. Donal O'Shea. Healthy bodyweight controls (BMI ≤ 25) were recruited from St. Vincent’s University Hospital.

Demographic data of the cohorts included in this study are presented in Supplementary Tables 1–5.

### Blood sampling, collection and storage

Blood samples were processed fresh and within 2 h. Blood was collected into EDTA-containing tubes and centrifuged at room temperature at 400 g for 10 min. The upper phase was transferred to a fresh tube and centrifuged at 2000*g* for 10 min. Plasma was then aliquoted and stored at − 80 °C.

### Gene expression in PBMCs, CD4^+^ T cells and CD4^−^ cells

Peripheral blood mononuclear cells (PBMCs) were isolated from whole blood in 2% foetal bovine serum in PBS using Ficoll-Paque (GE Healthcare, 17-1440-02) in Sepmate tubes (StemCell Technologies). CD4^+^ T cells were then isolated using StemCell Technologies RoboSep™ Human CD4^+^ T Cell Isolation Kit (Cat. No. #17952RF) according to the manufacturer’s instructions. RNA was extracted using Trizol reagent (Thermo Fisher) and assessed for purity and concentration by Nanodrop spectrophotometry. Samples were treated with DNase I (Invitrogen) before reverse transcription with Superscript IV (Invitrogen) as described previously [1]. qPCR was performed using Taqman Universal PCR Mastermix (Thermo Fisher) and probes for *CCN3* (Thermo Fisher; Hs00159631_m1) and *SDHA* (Hs00188166_m1) as a reference gene. Gene expression changes are reported as fold changes relative to healthy controls using the 2^−ddCt^ method.

### Measurement of CCN3 by ELISA

CCN3 levels in biological fluids and T cell culture supernatants were assessed by ELISA according to the manufacturer’s instructions (DY1640 DuoSet kit, R&D Systems). Standard curves were generated using four-parameter logistic curve-fit.

### Immunoblotting of CCN3

Plasma, CSF or h295-R cell-conditioned media were enriched for CCN3 using heparin-sepharose beads (GE Healthcare, #17-0998-01) as previously described [35]. In short, up to 4 ml (equivalent to at least 20 ng of CCN3) was added to 20 μl 50% heparin-sepharose slurry and incubated on a rotator at 4 °C overnight. In total, 25 μl of PBS-washed CCN3-bound heparin-sepharose beads and 5 μl of 6× reducing loading dye were boiled for 10 min prior to loading on a reducing 15% SDS-PAGE. Following transfer onto PVDF membrane (Millipore) and blocking (3% BSA in PBS/1% Tween) for 1 h at room temperature, protein was probed using polyclonal goat anti-human CCN3 antibody (0.1 mg/ml; cat. no. AF1640, R&D Systems) overnight at 4 °C and secondary rabbit anti-goat HRP (1:2000; #61-1620) for 1.5 h at room temperature. Bands were detected by chemiluminescence using Pierce™ ECL Western Blotting Substrate (Thermo Fisher, #32106) and imaged using a G:BOX detection system (Syngene).

### MS lesion characterisation and analysis

Immunohistochemistry was carried out on clinically and neuropathologically validated paraffin wax-embedded tissue from the UK Multiple Sclerosis brain bank at Imperial College London (research ethics approval 08/MRE09/31+5). All cases of MS and non-neurological controls had a death to preservation time of < 24 h, with the exception of control 03 (Ctrl03; death to preservation time of 36 h; Supplementary table 5). One or two pathological blocks containing cortical grey and subcortical white matter (superior frontal or cingulate cortex) were selected per case following tissue characterisation. Tissue sections were stained with Luxol-Fast Blue (LFB) and immunohistochemically stained with anti-proteolipid protein (PLP) and anti-CD68 to characterise areas of inflammatory demyelination or remyelination in the grey and white matter. White matter lesions were defined as active (demyelinated lesion with abundant CD68^+^ microglia/macrophages throughout the lesion and the presence of myelin (PLP^+^) degradation products); chronic active (abundant CD68^+^ microglia/macrophages restricted to the lesion edge and the presence of myelin degradation products) or chronic inactive (small numbers of mostly ramified CD68^+^ microglia at the well-defined lesion edge). Fully remyelinated white matter lesions were classified as shadow plaques based on the ‘normally appearing’ PLP profile and classic LFB ‘shadow’ appearance. Grey matter lesions were characterised based on their location, and subpial cortical grey matter lesions (clear ribbons of subpial cortical demyelination extending partly through the depth of the cortex), being the most common cortical grey matter lesion type in acute and progressive MS [39], were selected for analysis. Putative areas of cortical grey matter remyelination were defined based on a reduced density of PLP^+^ myelin sheaths and the presence of numerous PLP^+^ oligodendrocyte cell bodies per microscopic field of view [40].

### Immunostaining

Briefly, following dewaxing in xylene and rehydration through a graded series of ethanol, slides from MS and control cases were heated in a 0.05% solution of citraconic anhydride (Sigma; for immunohistochemistry) or sodium citrate (pH 6) for subsequent immunofluorescence cytochemistry. Endogenous peroxidases were quenched with a 0.6% hydrogen peroxide before a serum block. Slides were then incubated with primary antibody overnight (Table 1). Slides were washed, and relevant species-specific biotinylated secondary antibody (raised in horse or goat; Vector Laboratories) was applied. For immunohistochemistry, antibody binding was visualised with the avidin-biotin-complex (ABC) peroxidase-linked reporter system, with 3,3′-diaminobenzidine (DAB; Immpact DAB; Vector) as chromogen. Tissue sections were counterstained with Gill’s haematoxylin No. 2 (GHS232; Sigma) and mounted with DPX. Please note that for quantitative analysis of CCN3^+^ immunoreactivity all slides were stained in the same experimental run. For dual antibody immunofluorescence, bound primary antibody 1 (anti-human nuclear antigen (HuC)) was detected with an Alexa-488 conjugated anti-mouse secondary and the sequentially added primary antibody 2 (anti- CCN3) detected with an Alexa-546 anti-goat secondary. Slides for immunofluorescence were then quenched with 0.05% Sudan Black B to reduce autofluorescence before mounting in 12.5% mowiol/glycerol with DABCO. Negative controls were included, and in all instances, the relevant IgG control, or the absence of the secondary detection antibody, yielded no signal.

**Table 1:** Antibodies used for immunohistochemistry

Antibody (anti-)	Target	Source	Type/clone	Dilution
PLP	Proteolipid protein myelin	Abd Serotec, USA	mAb/plpc1	1:2000
CD68	Sialomucin, microglia and macrophages	DAKO, Denmark	mAb/KP1	1:400
CCN3	Human CCN3 protein	R&D Systems	pAb (AF1640)	1:200
HuC/D	Human HuC/HuD neuronal protein	Thermo Fisher Scientific	mAb/16A11	1:2000

Primary antibodies used in this study

### In situ hybridisation

In situ hybridisation (ISH) was performed using a 5 fluorescein (FAM)-labelled 22mer antisense oligonucleotide containing locked nucleic acid (LNA) and 2-O-methyl (2-O-Me)-RNA moieties in a 1:2 ratio (Eurogentec, Southampton, UK). Probe design was as follows: antisense CCN3 (NM_002514) FAM–CauCucAcaUugAcgGuuCcuA and sense FAM–TagGaaCcgTcaAtgTgaGatG (capitals indicate LNA and lowercase 2-O-Me-RNA). Following dewaxing, rehydration and antigen retrieval, sections were pre-hybridised in hybridization mix [41] before probe hybridization (30 min) in the same solution. Sections were then washed in decreasing concentrations of saline-sodium citrate buffer, washed in PBS, incubated with a peroxidase conjugated goat anti-FAM (Vector Labs) and visualised with DAB. Sections were counterstained with haematoxylin before dehydrating, clearing and mounting with DPX.

### Image acquisition and QuPath analysis

Immunohistochemically stained tissue sections for PLP, CD68 and CCN3 were digitised by whole slide-scanning using a Leica SCF400F scanner and handled using QuPath [42]. Areas of chronic active white matter demyelination and subpial cortical grey matter lesions, alongside areas of remyelinated white and grey matter, were first traced using the annotation tool on the PLP-stained slide for each section. The annotation was then transferred to the same location on the sequential CCN3-stained slide from the same case and the area fraction (% CCN3^+^ immunoreactivity) of the entire lesion or normal-appearing region of interest calculated.

### Statistical analyses

Analyses were performed with Microsoft Excel and GraphPad Prism. Normality of datasets was determined using the D’Agostino and Pearson test. Parametric datasets were tested for statistical significance using two tailed *t*-tests to compare two groups or one-way analysis of variance (ANOVA) with Tukey’s post hoc test for more than two groups. Mann-Whitney *U* tests or Kruskal Wallis tests with Dunn’s post hoc test were used for non-parametric data as appropriate. Comparisons between matched samples during relapse or remittance, or pre- and post-liraglutide were tested with Wilcoxon matched-pairs signed rank test. Correlation analyses were performed using the Pearson test for parametric data or the Spearman test for non-parametric data. Data are presented as means and standard error of the mean (SEM) unless otherwise stated. * = *p* < 0.05. ** = *p* < 0.01, *** = *p* < 0.001.

### Data availability

Anonymised datasets used and/or analysed during the current study are available from the corresponding author on reasonable request.

## Results

### Plasma CCN3 levels are altered by MS disease phase and treatment

**Fig. 1:**
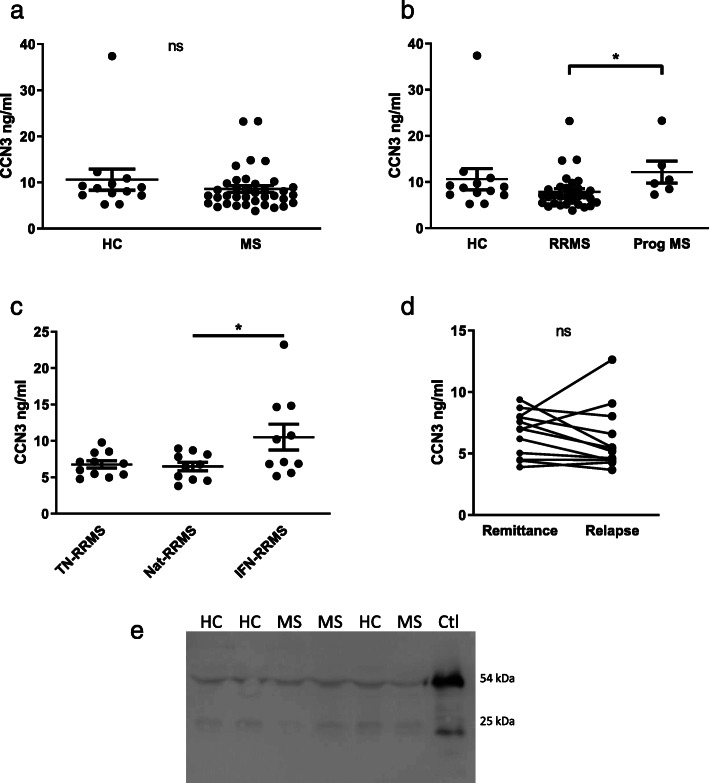
Plasma CCN3 levels are altered by MS disease phase and treatment. Plasma CCN3 levels are compared in healthy controls and MS (**a**), controls and MS disease phase (**b**), RRMS treatment groups (**c**) and matched samples from RRMS patients during relapse or remittance (**d**). Detection of plasma-derived CCN3 by western blot (**e**). Abbreviations: HC, healthy control; MS, multiple sclerosis; RRMS, relapsing-remitting MS; Prog MS, progressive MS; TN, treatment-naïve; Nat, natalizumab treatment; IFN, interferon-β treatment; Ctl, h295-R-conditioned medium as positive control

To assess whether CCN3 levels were altered in MS, plasma samples from patients with MS or age- and sex-matched healthy controls (HC) were analysed by ELISA. Mean CCN3 plasma concentration did not differ between groups (MS; 8.58 ± 4.45 ng/ml; HC 10.61 ± 8.29 ng/ml, Fig. 1a), but significantly higher CCN3 levels were observed in patients with progressive MS (PrMS) versus RRMS (PrMS; 12.14 ± 5.86 ng/ml; RRMS 7.89 ± 3.87 ng/ml, Fig. 1b). A significant effect of treatment was detected in RRMS patients. Post hoc tests detected significantly elevated levels of CCN3 in patients on interferon-β versus those on natalizumab (IFN-β; 10.52 ± 5.66 ng/ml, natalizumab; 6.49 ± 1.87 ng/ml, Fig. 1c). Matched samples were analysed from MS patients during relapse and remitting phases of disease, but no difference in plasma CCN3 was detected (Fig. 1d). To concentrate samples for detection by western blot, plasma samples were incubated on heparin-sepharose beads to which CCN3 binds through the C-terminal domain. Two major CCN3 isoforms were detected in plasma of both MS patients and healthy controls at approximately 54 kDa and 25 kDa, likely representing full-length CCN3 and the amino-truncated isoform detected in the control cell culture-conditioned medium of h295-R cells expressing CCN3 [43] (Fig. 1e).

### Obesity-associated changes in plasma CCN3 levels are evident in MS

**Fig. 2:**
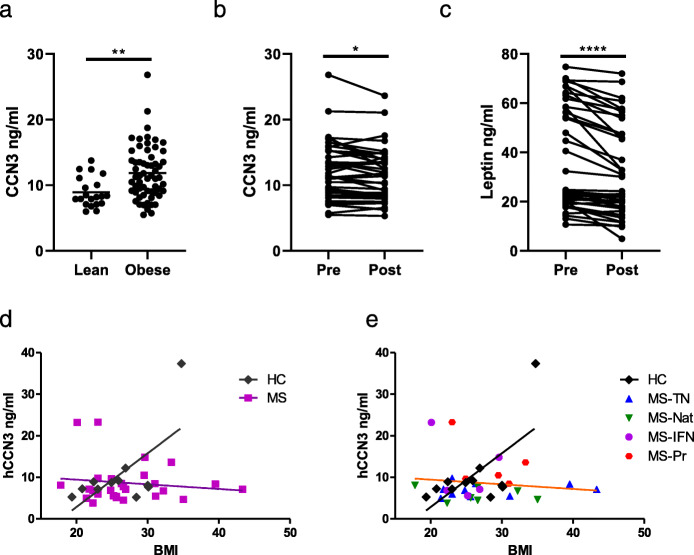
Obesity-induced changes in plasma CCN3 levels are evident in MS. Plasma CCN3 levels in lean vs. obese cohorts (**a**), CCN3 levels (**b**) and leptin levels (**c**) in plasma from obese patients before and after 12 weeks of GLP-1 agonist therapy. Corresponding plasma CCN3 levels and BMI in healthy controls and MS (**d**) and stratified by disease phase and treatment (**e**). Abbreviations: HC, healthy control; MS, multiple sclerosis; RRMS, relapsing-remitting MS; TN, treatment-naïve; Nat, natalizumab treatment; IFN, interferon-β treatment; MS-Pr, progressive MS

Increasing body mass index (BMI) was reported to strongly correlate with increasing plasma CCN3 levels [44]. To validate this finding, serum CCN3 concentrations were analysed from obese patients and healthy bodyweight (‘lean’) controls who did not have MS. This revealed significantly raised levels of CCN3 in groups in which BMI differences ranged from 25 kg/m^2^ between lean and obese cohorts, to just 1–5 kg/m^2^ in matched samples from patients pre- and post-treatment with the glucagon-like peptide (GLP)-1 agonist, liraglutide (Fig. 2a,b). This mirrored changes observed in leptin, another adipokine, in this cohort (Fig. 2c). Linear regression analysis confirmed a significant relationship between BMI and CCN3 plasma levels in age- and sex-matched healthy controls, but this relationship was absent in MS patients (Fig. 2d,e; HC; *r*^2^ = 0.427, slope = 1.294 ± 0.50, *p* = 0.029 MS; *r*^2^ = 0.018, slope = − 0.113 ± 0.17, *p* = 0.505). This effect was most prominent in obese patients with MS (BMI > 30), and this effect had no clear association to any treatment group or progressive disease (Fig. 2e).

### *CCN3* mRNA is significantly upregulated in peripheral immune cells in MS

CCN3 has previously been shown to be secreted by polarised murine T cells in vitro [1]. While we occasionally observed detectable CCN3 from human CD4^+^ T cell culture-conditioned medium, it was not consistent across donors or conditions and was very close to the limit of detection (data not shown).

**Fig. 3:**
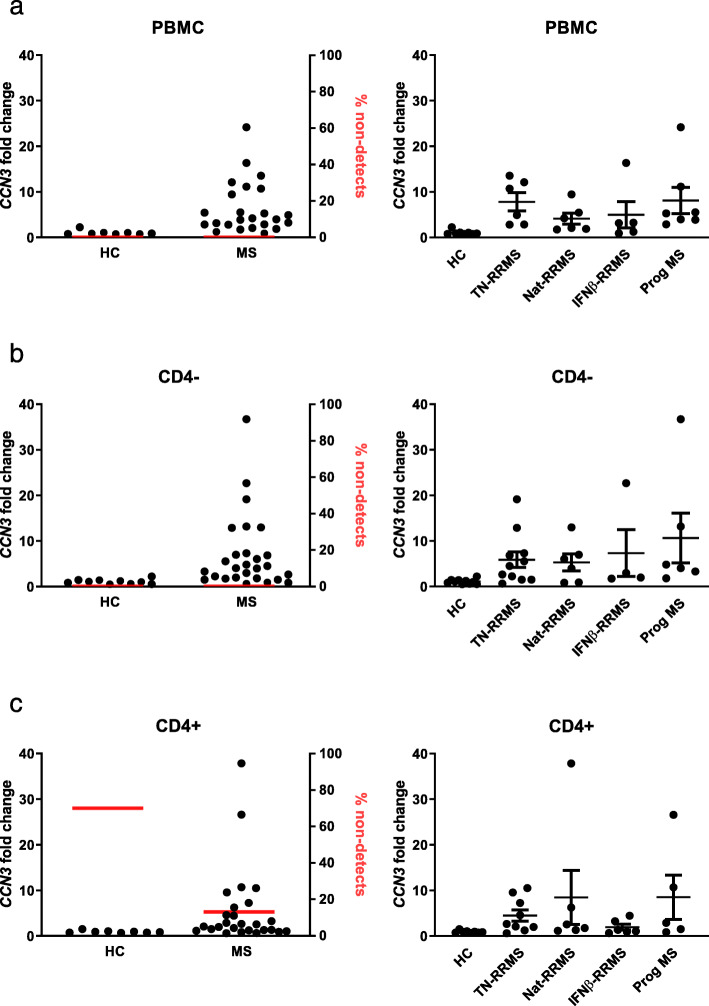
*CCN3* is significantly upregulated in peripheral immune cells in MS. Fold change in *CCN3* gene expression in PBMCs (**a**), CD4^−^ (**b**) and CD4^+^ (**c**) peripheral immune cells in MS relative to age- and sex-matched healthy controls. The percentages of samples with detectable expression are indicated in red (left graphs, **a**–**c**). *CCN3* mRNA expression levels in healthy controls and MS patients stratified by disease phase and treatment (right graphs, **a**–**c**). Abbreviations: HC, healthy control; MS, multiple sclerosis; RRMS, relapsing-remitting MS; TN, treatment-naïve; Nat, natalizumab treatment; IFN, interferon-β treatment; Prog MS, progressive MS

We asked whether regulation of *CCN3* gene expression by immune cells is altered in MS. *CCN3* mRNA expression was upregulated in PBMCs of MS versus matched controls and in both CD4^−^ and CD4^+^ immune cells as determined by qPCR (Fig. 3a–c). While only one third of samples from healthy controls had detectable expression of *CCN3* mRNA in the CD4^+^ cell population, 87% of samples from MS patients had detectable expression (Fig. 3c). *CCN3* expression was notable in all disease and treatment groups tested. No correlation was observed between *CCN3* gene expression by peripheral immune cells and plasma concentration (data not shown).

### Significant correlation between plasma and CSF levels of CCN3 in MS

**Fig. 4:**
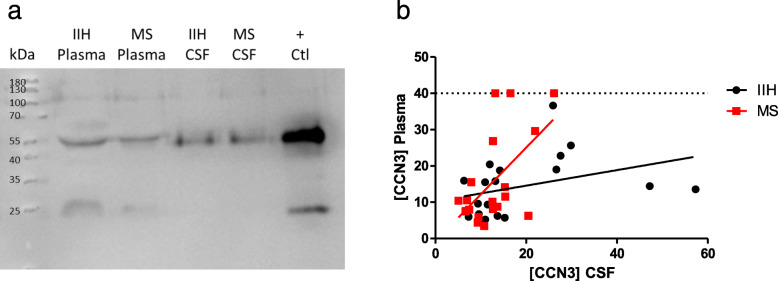
Significant correlation between plasma and CSF levels of CCN3 in MS. Western blot of CCN3 in matched plasma and CSF of MS subtypes and IIH controls (**a**). Relationship between plasma CCN3 and CSF CCN3 levels in IIH patients (black) and MS patients (red) (**b**). Abbreviations: CSF, cerebrospinal fluid; IIH, idiopathic intracranial hypertension; MS, multiple sclerosis; +Ctl, h295R-conditioned medium

Although CCN3 has previously been detected in human CSF [36], the physiological concentration range and relationship to plasma levels had not been established. To address whether these parameters change in MS, we analysed matched plasma and CSF samples from RRMS patients who had lumbar puncture performed for diagnosis. This was compared to an age- and sex-matched cohort of patients with idiopathic intracranial hypertension (IIH) who underwent CSF shunting to relieve pressure on the brain. Identical CCN3 isoforms were detected in plasma samples of IIH and MS patients as had been detected in healthy controls (Fig. 1e, Fig. 4a). In contrast to plasma, only the higher molecular weight isoform of CCN3 was detected in CSF and this was consistent in both IIH and MS CSF samples (Fig. 4a).

CSF CCN3 levels were comparable between MS and IIH groups and there was no difference between means of plasma or CSF levels in either group (Fig. 4b). Pearson correlation analysis detected a significant correlation between increasing plasma and CSF concentration levels in MS patients which was absent in IIH samples (Fig. 4b, IIH: *r*^2^ = 0.125, ns; MS: *r*^2^ = 0.335, *p* < 0.01).

**Fig. 5:**
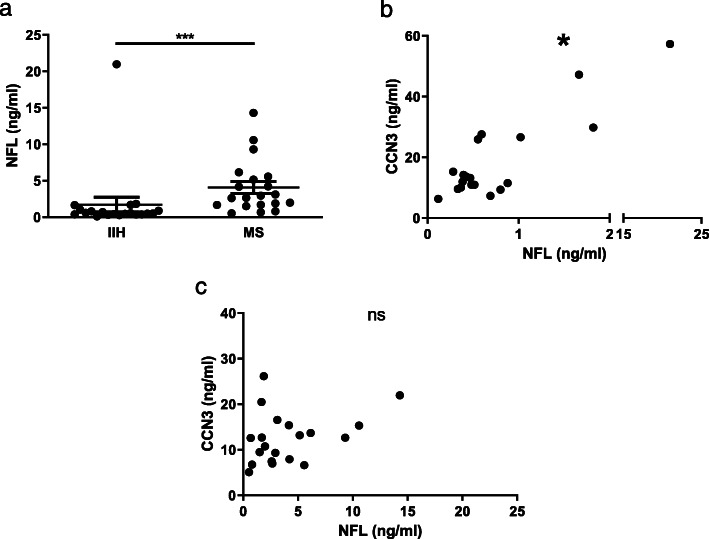
Neurofilament levels and CCN3 levels correlate in CSF of IIH patients but not MS. Neurofilament light (NFL) levels in CSF of matched IIH and MS donors (**a**). Relationship between CCN3 levels and NFL levels in CSF of IIH patients (**b**) and MS patients (**c**). Abbreviations: CSF, cerebrospinal fluid; IIH, idiopathic intracranial hypertension; MS, multiple sclerosis; NFL, neurofilament light

To determine whether CSF CCN3 levels were related to neuropathological processes, we analysed expression relative to CSF concentrations of neurofilament light (NFL) chain protein, a marker of axonal degeneration. As expected, MS CSF had higher NFL levels compared to IIH CSF (Fig. 5a). The Spearman analysis identified a significant correlation between increasing CCN3 and NFL concentration levels in CSF of IIH patients (Fig. 5b), but this was not the case in MS samples (Fig. 5c, IIH: *r* = 0.532, *p* < 0.05; MS: *r* = 0.326, ns). This suggests that more complex, parallel processes lead to a non-linear relationship in MS.

### Area of CCN3 immunoreactivity is not significantly different across MS lesions

**Fig. 6:**
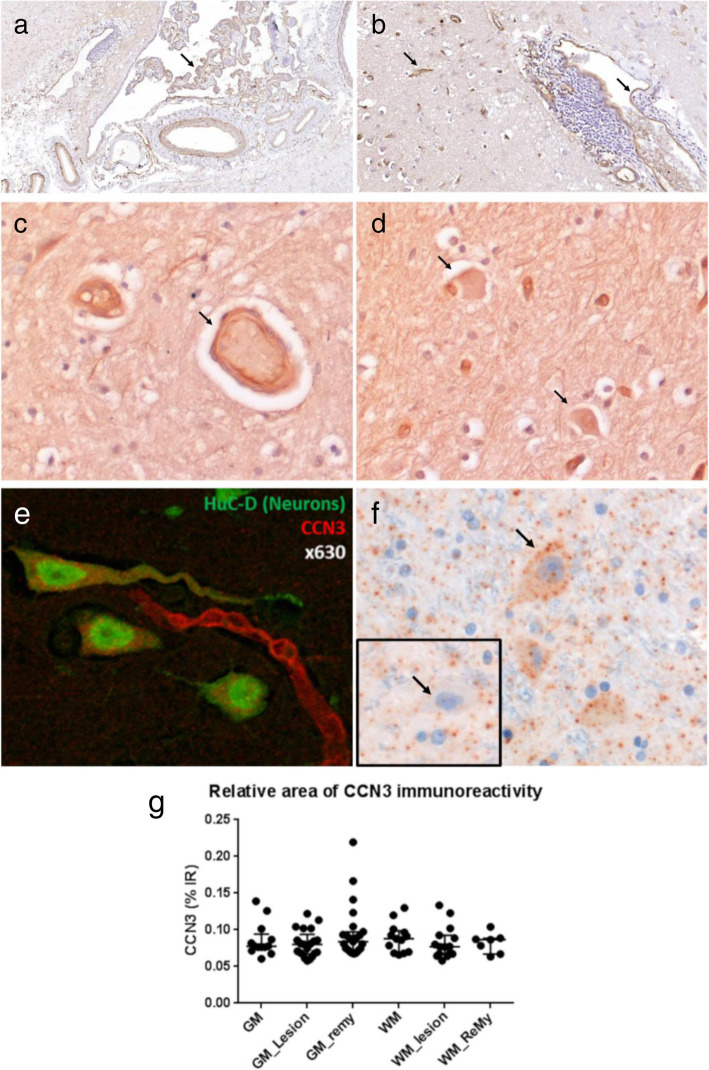
CCN3 immunostaining in normal and MS brain tissue. CCN3 is present in blood vessels, choroid epithelia and neuron-, and macroglia-like cells of the CNS (black arrows, **a**–**d**). Co-labelled sections of MS grey matter with antibodies to the mature neuronal protein HuC/D (green) and anti-CCN3 (red) demonstrates dual-labelling of CCN3 and HuC/D^+^ neurons (**e**). Detection of CCN3 transcripts by in situ hybridisation in MS grey matter with a distinct neuronal morphology (**f**) and respective negative control (**f**, inset). Area of CCN3 immunoreactivity in MS brain tissue (*n* = 17 cases) (**g**). GM, normal-appearing grey matter; GM Lesion, demyelinated GM, GM remy, remyelinated GM; WM, normal-appearing white matter; WM Lesion, demyelinated WM; WM remy, remyelinated WM

Immunohistochemistry (IHC) revealed CCN3 protein expression most consistently associated with adluminal faces of arteries and vessels in both healthy control and MS brain tissue (Fig. 6a,b). Large arteries and veins in the subarachnoid space, connective tissue of the meninges and pia, and epithelia of the choroid plexus displayed anti-CCN3 immunoreactivity. Within the brain parenchyma, cells resembling neurons and macroglia were anti-CCN3^+^ (Fig. 6c,d). Specific neuronal anti-CCN3 immunoreactivity was confirmed by dual immunofluorescence with the neuron-lineage antigen HuC/D (Fig. 6e) and by the detection of CCN3 transcript in large neuron-like cells with a pyramidal morphology of the cerebral cortex (Fig. 6f). To understand the relationship between relative CCN3^+^ immunoreactivity and MS pathology, we calculated the percent area of specific anti-CCN3^+^ signal for normal-appearing, demyelinated, and remyelinated white and grey matter, respectively, from brain tissue blocks from 17 cases. The extent of CCN3^+^ immunoreactivity was highly variable, with, for example, cases of cortical grey matter remyelination presenting with > 15% of plaque comprising CCN3^+^ structures. Nevertheless, the mean extent of CCN3^+^ immunoreactivity was unchanged between any of the MS regions of interest analysed (*p* > 0.9, Fig. 6g).

## Discussion

We recently uncovered CCN3 as a key mediator of neuroimmune communication between T cells and glia in the murine central nervous system [1]. Treg-derived CCN3 promoted oligodendrocyte differentiation and myelin production in murine in vitro and ex vivo models. As this could have direct relevance for people with demyelinating diseases such as MS, we wanted to determine whether levels of CCN3 should be targeted therapeutically. Evidence to support this approach would be a deficit of CCN3 systemically (in plasma or CSF), locally (in lesion tissue) or potentially key cellular sources (PBMCs, T cells). In this study, we observed no difference in CCN3 levels in plasma or CSF between people with MS and controls indicating that there is no systemic deficit to be addressed in MS. Furthermore, we observed no difference in CCN3 levels between demyelinated and remyelinated CNS lesions suggesting that CCN3 production is not a limiting factor constraining myelin repair. Despite comparable systemic levels, CCN3 was significantly upregulated in PBMCs and T cells of MS patients and almost absent in control samples, suggesting that cellular sources of CCN3 may vary as a result of MS but overall levels are not altered. This study shows that although our previous studies showed that CCN3 can potentiate oligodendrocyte differentiation, there is no paucity of CCN3 production that needs to be addressed to boost myelin repair in MS. Indeed recently published work by our group shows that CCN3-deficient mice can efficiently regenerate myelin in mouse models of remyelination [32]. While there are still no remyelinating therapies available for MS, with clinical trials only beginning in recent years, we believe that it is important to the progress of the field to report the follow-up of potential targets, even when, and indeed particularly when, no overt differences are observed.

Though our study may be negative in this respect, it points to other functions of this protein whose roles in terms of immune function and regeneration are an active area of research and which has been relatively unstudied in the CNS or multiple sclerosis. Our data can help provide clinical context and direction as this research unfolds.

As in mice, we observed that activated human CD4^+^ T cells secreted CCN3 in vitro. However, CCN3 levels were near the limit of detection of the ELISA and would not be sensitive enough to detect differences at that range. Analysis of polarised T cells from mice suggests that expression of CCN3 is subset specific (data not shown). Gene expression analysis of CD4^+^ T cells and PBMCs revealed stark differences between MS patients and controls. In contrast to CCN3 expression in MS samples, control samples had consistently low *CCN3* expression in PBMCs and low positive detection rates in CD4^+^ T cells. This mirrors *CCN3* gene expression analysis of whole blood from healthy populations reported elsewhere [45]. The functions of CCN3 in peripheral immune cells are not well understood. CCN3 has been identified as a critical regulator of human haematopoietic stem cells from umbilical cord blood [17, 18]. Through increasing glycolytic enzymes and manipulating stem cell state, soluble CCN3 dramatically increased the number of serially transplantable cells. Preliminary work indicated that CCN3 could stimulate mobilised adult CD34^+^ cells in similar ways [17]. In MS patients treated with natalizumab, persistent MRI and clinical disease activity occurred in patients that lacked HSC mobilisation post-treatment [46]. Understanding the role of CCN3 in adult HSC and peripheral immune cells could reveal important insights for MS.

Plasma levels of CCN3 reflect many cellular sources including adipose tissue and are known to correlate to increasing BMI and obesity [20, 44]. No differences were observed in plasma levels of CCN3 between MS and HC. Further analysis confirmed a positive correlation of CCN3 to BMI in the control cohort, but surprisingly, there was no such correlation in the larger MS cohort. Far from just an energy reservoir, adipose tissue exerts systemic effects on endocrine and immune systems, primarily mediated by adipokines. CCN3 is expressed by adipocytes and is believed to contribute to obesity-related inflammation and conditions including type 2 diabetes, sleep apnoea and obesity-induced cardiomyopathy [20, 44, 47, 48]. CCN3 is upregulated in adipose tissue when mice are fed a high-fat diet whereas CCN3-deficient mice gain less weight and have better glucose tolerance and insulin sensitivity [19, 44]. Less inflammation was observed in the adipose tissue of CCN3-deficient mice including significantly lower percentages of CD4^+^ T cells. Our finding suggests that the strong association between BMI and plasma CCN3 observed in controls and in other studies diverges in MS patients and this is most prominent in patients with high BMI. Obesity, particularly at a young age, is an established risk factor for MS [49–53], and differences in adipokine regulation may prove important to disease pathogenesis [54].

Plasma levels of CCN3 were not altered during MS relapse. In addition, plasma levels had no correlation to EDSS, number of relapses in the past year or disease duration (data not shown). This is in contrast to rheumatoid arthritis, where higher serum CCN3 was associated with higher disease activity and inflammatory markers [55]. Despite this, we observed that CCN3 was raised in progressive MS compared to RRMS and dynamically regulated by disease-modifying treatment. Plasma CCN3 levels were significantly higher in patients on interferon-β treatment versus natalizumab. This is intriguing as both therapeutics decrease MS disease activity (CNS inflammatory lesions detected by MRI) and relapse rates but by distinct mechanisms; IFN-β exerts immunoregulatory effects on a range of immune cells such as T cells and antigen-presenting cells whereas natalizumab directly blocks T cell migration from the vasculature into the CNS [56, 57]. In addition, interferon-β treatment appeared to block the upregulation of CCN3 in CD4^+^ T cells detected in all other MS patient groups. Interferon-β can also act directly on adipocytes suggesting that interferon-β may potentially have independent effects on CCN3 expression by immune cells and adipocytes.

A positive correlation was detected between plasma and CSF CCN3 levels in MS which was absent in IIH patients. This could be a result of passive protein level changes associated by blood brain barrier breakdown in MS [58], and/or a more actively induced change by infiltration of peripheral immune cells into the CNS compartment. Interestingly, the CCN3 isoforms identified in blood differed from CSF. Whereas full-length CCN3 was detected in both compartments, serum samples also contained a shorter isoform around 25 kDa, similar in size to the previously reported amino-truncated CCN3 species [3, 7, 8, 26, 36, 43, 59]. This may be due to differentially regulated gene expression, post-translational mechanisms or even different degradative processes in these compartments [8]. Truncated isoforms of CCN3 are suggested to be developmentally regulated and have previously been detected in brain tissue [26, 60].

In the CNS, we detected CCN3 expression in neurons, blood vessels, astrocytes and choroid epithelia, demonstrating that CCN3 can be produced by a range of neural cell types. This is similar to reports of CCN3 expression in mouse, though detection of CCN3 in blood vessels and choroid epithelia has not been described in the murine CNS. CCN3 was upregulated upon toxin-induced demyelination and specifically expressed in GFAP^+^ cells and oligodendroglia within remyelinating lesions, although it was not essential for efficient remyelination to take place [32]. This would suggest that Treg-derived CCN3 is not the principal cellular source of CCN3 in the CNS particularly given the low density of Treg in the CNS compared to neural cell types. Importantly, overall CCN3 expression was similar in demyelinated, remyelinated and normal-appearing tissue in the grey and white matter of MS brain tissue. This suggests that a deficit of CCN3 production is unlikely to be a factor limiting myelin repair in MS lesions. The diverse neural sources of CCN3 also suggest that CCN3 likely exerts a range of biological functions beyond oligodendrocyte differentiation, and indeed, expression in normal CNS tissue suggests that CCN3 may fulfil key roles in CNS homeostasis.

Though our previous work showed that Treg-derived CCN3 was a positive mediator of oligodendrocyte differentiation in vitro and ex vivo, comprehensive follow-up was required to validate whether CCN3 would be an appropriate target for remyelinating therapy. Our analysis within the CNS, CSF or plasma suggests there is no systemic deficiency of CCN3 to be targeted in MS. Recent work from our group demonstrated that murine remyelination is still efficient in the absence of CCN3 [32]. This does not exclude the possibilities that binding partners and signalling mediators of CCN3 could still contribute to myelin repair. Functional mechanisms of CCN3 in the CNS are still unknown and require further study. Nonetheless, this work has highlighted dynamic regulation of this multifaceted matricellular protein and adipokine in MS that will be informative for future studies. Given the known roles and effects of CCN3 in haematopoiesis and as an adipokine modulator of immune function, it remains relevant to understand its expression profile particularly considering the pathogenic role of both obesity and the adaptive immune system in multiple sclerosis. The robust expression of CCN3 in CNS and CSF also points to functions that have yet to be uncovered. Our study provides a comprehensive characterisation of CCN3 in the periphery and CNS. This revealed dynamic profiles of CCN3 in MS disease phase and treatment, and pointed to differential regulation as an adipokine in patients with high BMI. However, the overarching finding of comparable systemic levels of CCN3 in samples from healthy controls and people with MS does not provide rationale to broadly supplement CCN3 therapeutically in MS.

## Conclusions

We analysed CCN3 in distinct cohorts to establish physiological ranges and determine whether CCN3 is altered by disease state in peripheral and in CNS compartments. Ultimately, the robust expression of CCN3 in the periphery and CNS of individuals with MS shows that there is not an obvious CCN3 deficiency in MS that should be directly targeted therapeutically, but its altered profile of expression potentially points to additional cellular players, functions and regulatory mechanisms. Whether therapeutic potential remains in the functional realm of CCN3 biology will require future studies of human CCN3 in neural functional studies. Crucially, the studies described here that provide a comprehensive characterisation and comparative analysis of CCN3 in MS and in different anatomical compartments will underpin such future research efforts in this area.

## Supplementary Information

**Additional file 1.** Supplementary tables 1-5.

**Additional file 2.** Fig. 4a raw image of western blot.

## Data Availability

Anonymised datasets used and/or analysed during the current study are available from the corresponding author on reasonable request.

## Abbreviations

BMI: Body mass index
BSA: Bovine serum albumin
CCN3: Cellular communication network factor 3
Ctl: Control
DAB: 3,3′-Diaminobenzidine
ECL: Enhanced chemiluminescence
EDTA: Ethylenediaminetetraacetic acid
ELISA: Enzyme-linked immunosorbent assay
GLP: Glucagon-like peptide
GM: Grey matter
HC: Healthy control
HRP: Horseradish peroxidase
HSPG: Heparin sulphate proteoglycan
HuC: Human nuclear antigen
IFN-β: Interferon-β treatment
IGFBP: Insulin-like growth factor binding protein
IIH: Idiopathic intracranial hypertension
IHC: Immunohistochemistry
LFB: Luxol-Fast Blue
LNA: Locked nucleic acid
MS: Multiple sclerosis
Nat: Natalizumab treatment
NFL: Neurofilament light
PBS: Phosphate-buffered saline
PBMCs: Peripheral blood mononuclear cells
PrMS: Progressive MS
PLP: Proteolipid protein
PVDF: Polyvinylidene fluoride
remy: Remyelinated
RRMS: Relapsing remitting MS
SDS-PAGE: Sodium dodecyl sulphate-polyacrylamide gel electrophoresis
TN: Treatment-naïve
Treg: Regulatory T cells
TSP1: Thrombospondin type I repeat
VWC: von Willebrand factor type C repeat
WM: White matter
