# Supplementary material for: CCN3 is dynamically regulated by treatment and disease state in multiple sclerosis
Source: J Neuroinflammation. 2020 Nov 22;17:349. doi: 10.1186/s12974-020-02025-7 (PMC7681974; doi:10.1186/s12974-020-02025-7)
Supplement: Supplementary file 1 — Additional file 1. Supplementary tables 1-5. [file 12974_2020_2025_MOESM1_ESM.pdf]

Additional File 1

Supplementary Table 1

MS	Diagnosis	Treatment	Gender	Age	BMI
A01	RRMS	Treatment-naïve	F	33	21.4
A02	RRMS	Treatment-naïve	M	52	31.1
A03	RRMS	Treatment-naïve	M	45	39.5
A04	RRMS	Treatment-naïve	F	37	N/A
A05	RRMS	Treatment-naïve	F	62	25.6
A06	RRMS	Treatment-naïve	M	47	24.8
A07	RRMS	Treatment-naïve	F	42	43.3
A09	RRMS	Treatment-naïve	F	59	21.8
A10	RRMS	Treatment-naïve	F	38	23
A11	RRMS	Treatment-naïve	F	72	23
A12	RRMS	Treatment-naïve	F	45	26.3
B01	RRMS	Natalizumab	F	32	N/A
B02	RRMS	Natalizumab	F	40	N/A
B03	RRMS	Natalizumab	M	36	N/A
B04	RRMS	Natalizumab	F	58	25.6
B05	RRMS	Natalizumab	F	54	32.2
B07	RRMS	Natalizumab	M	47	26.6
B08	RRMS	Natalizumab	F	33	26.6
B09	RRMS	Natalizumab	M	44	17.8
B10	RRMS	Natalizumab	M	33	35
B11	RRMS	Natalizumab	M	39	22.3
C01	RRMS	Interferon-beta	F	N/A	N/A
C02	RRMS	Interferon-beta	F	55	N/A
C03	RRMS	Interferon-beta	F	54	N/A
C04	RRMS	Interferon-beta	F	57	N/A
C05	RRMS	Interferon-beta	F	52	22.3
C06	RRMS	Interferon-beta	F	37	20.1
C07	RRMS	Interferon-beta	F	43	26.9
C08	RRMS	Interferon-beta	M	64	25.2
C09	RRMS	Interferon-beta	F	31	N/A
C10	RRMS	Interferon-beta	F	36	29.6
D01	SPMS	-	F	51	23
D02	SPMS	-	M	58	29.5
D03	SPMS	-	F	64	24.9
D04	SPMS	-	F	54	N/A
D05	SPMS	-	F	80	33.3
E01	PPMS	-	M	56	31
			26F 11M	48.3±11.8	27.1±5.8

Heatlhy controls			Gender	age	BMI

H05	-	-	F	56	N/A
H08	-	-	F	46	20.8
H09	-	-	F	63	22.4
H10	-	-	F	48	26.9
H11	-	-	F	50	23
H12	-	-	F	39	30.1
H14	-	-	M	41	30
H15	-	-	F	38	34.72
H16	-	-	M	37	25.9
H17	-	-	F	41	28.4
N01	-	-	F	32	19.36
N03	-	-	F	25	N/A
N06	-	-	M	47	24.9
			10F 3M	43.3±9.6	26.0±4.3

Supplementary Table S1: Clinical characteristics of multiple sclerosis patients and healthy controls recruited at QUB/BCH for plasma and immune cell gene expression analysis.

Supplementary Table 2

ID	Diagnosis	Age (y)	Gender
17820	IIH	23.7	F
18771	IIH	39.0	M
21758	IIH	27.1	F
24414	IIH	43.8	F
24939	IIH	47.3	F
24943	IIH	26.7	F
25011	IIH	33.5	F
25030	IIH	51.8	M
25204	IIH	28.1	F
25220	IIH	26.2	F
25229	IIH	31.0	M
25272	IIH	30.0	F
25308	IIH	21.2	F
26574	IIH	37.4	F
25498	IIH	25.6	F
25562	IIH	27.6	F
26576	IIH	22.3	F
27110	IIH	20.4	F
26441	IIH	30.1	F
26506	IIH	43.2	M
	N=20	31.8±8.8	16F 4M
7204	RRMS	35.6	F
13368	RRMS	48.8	M
21510	RRMS	35.7	F

27835	RRMS	23.1	F
24946	RRMS	21.6	F
28012	RRMS	28.7	F
30277	RRMS	33.5	F
22789	RRMS	47.7	F
68722	RRMS	32.3	F
68399	RRMS	25.9	M
68773	RRMS	44.8	M
46249	RRMS	43.4	F
48749	RRMS	33.0	M
44151	RRMS	28.4	F
47658	RRMS	42.8	M
44914	RRMS	38.1	F
43475	RRMS	23.8	F
38076	RRMS	45.3	F
28122	RRMS	35.5	M
25969	RRMS	51.4	F
	N=20	36.0±8.9	14F 6M

Supplementary Table S2: Clinical characteristics of IIH and RRMS patients for matched plasma and CSF sample analysis.

Supplementary Table 3

Lean	Sex	Age	BMI	HbA1c
1	F	29	23	29
2	M	47	25	N/A
3	F	33	18	N/A
4	F	55	25	37
5	F	48	23	38
6	F	32	21	35
7	M	34	25	32
8	F	26	21	30
9	F	26	23	31
10	F	32	19	N/A
11	M	43	24	38
12	F	41	25	30
13	F	64	25	40
14	M	31	24	N/A
15	F	43	24	N/A
16	M	31	22	36
17	M	33	23	N/A
18	F	31	21	N/A
19	F	38	22	33
20	M	40	24	N/A

	13F 7M	37.9±9.4	22.9±1.9	

Obese	Sex	Age	BMI	HbA1c
1	M	42	49	35
2	F	44	50	36
3	F	44	48	33
4	F	41	58	31
5	M	44	38	42
6	M	54	63	40
7	F	54	49	39
8	F	57	43	37
9	F	42	33	40
10	M	57	47	55
11	M	50	42	48
12	F	37	64	36
13	F	38	42	37
14	F	58	46	85
15	F	38	42	42
16	F	60	46	58
17	F	37	59	61
18	F	56	62	N/A
19	M	61	47	41
20	F	42	42	42
21	F	44	48	42
22	M	49	44	40
23	M	60	39	40
24	M	54	45	69
25	F	53	37	46
26	F	58	37	55
27	F	40	43	N/A
28	F	25	46	39
29	F	35	51	N/A
30	F	47	40	131
31	M	54	49	72
32	F	27	64	42
33	F	38	46	40
34	F	44	63	41
35	M	57	48	60
36	F	32	52	31
37	F	46	57	34
38	F	28	44	36
39	F	30	51	40
40	F	48	46	40
41	M	57	44	42

42	M	18	56	37
43	F	60	44	47
44	M	45	33	32
45	F	62	51	49
46	F	27	45	32
47	F	43	48	N/A
48	M	42	45	35
49	M	35	46	42
50	F	47	39	38
51	F	34	49	N/A
52	F	28	40	37
53	F	44	52	39
54	F	50	71	36
55	F	42	50	32
56	M	46	69	41
57	F	58	46	93
58	F	34	55	44
59	F	42	47	37
60	F	52	55	50
	43F 17M	44.8±10.3	48.4±8.2	

Supplementary Table S3: Clinical characteristics of lean and obese cohorts used for CCN3 plasma analysis.

Supplementary Table 4

	Age	Gender
1	75.6	M
2	57.1	M
3	45.0	F
4	40.5	F
5	64.1	M
6	71.1	F
7	54.3	M
8	41.6	M
9	51.1	M
10	58.7	F
11	48.4	F
12	43.7	F
13	59.5	M
14	59.5	F
15	54.1	F
16	25.2	F
17	45.1	F
18	50.1	M
19	52.2	M

20	51.4	F
21	61.3	F
22	58.2	M
23	32.6	F
24	53.0	F
25	54.0	F
26	39.4	M
27	62.0	M
28	70.0	F
29	44.3	F
30	56.0	F
31	37.9	F
32	53.2	M
33	53.6	F
34	57.6	M
35	43.3	F
36	44.8	F
37	55.1	F
38	41.8	F
39	64.8	F
40	42.7	F
	51.8±10.4	26F 14M

Supplementary Table S4: Clinical characteristics of obese patients treated with GLP-1 agonist liraglutide.

Supplementary Table 5

Case	Gender	Type MS	Disease Duration	Age Died	Cause of death	No. blocks
**MS001**	M	SPMS	20	46	MS, bronchopneumonia	2
**MS002**	M	SPMS	25	62	MS, septicaemia,	1
**MS003**	F	SPMS	19	44	Septicaemia, pneumonia	1
**MS004**	M	SPMS	10	39	Pneumonia, sepsis	2
**MS005**	M	SPMS	26	61	Pancreatic Cancer	1
**MS006**	F	SPMS	30	54	Pneumonia	2
**MS007**	F	SPMS	21	46	Pneumonia	1
**MS008**	F	SPMS	18	53	MS	1
**MS009**	F	PPMS	13	39	MS, bronchopneumonia	1
**MS010**	F	PPMS	29	57	MS, bronchopneumonia	2
**MS011**	F	SPMS	29	60	Aspiration pneumonia	1
**MS012**	F	SPMS	22	38	MS, Pneumonia	1
**MS013**	M	SPMS	18	51	MS, respiratory failure	1
**MS014**	M	SPMS	25	47	MS, Pneumonia	1
**MS015**	F	SPMS	25	45	MS	2
**MS016**	M	SPMS	24	42	MS	2
**MS017**	M	SPMS	39	50	Pneumonia	1
**Ctrl01**	F	n/a	n/a	62	Colorectal cancer	1

**Ctrl02**	M	n/a	n/a	60	Breast cancer	1
**Ctrl03**	M	n/a	n/a	56	Cardiac arrest	1

Supplementary Table S5: Clinical characteristics of post-mortem cases for IHC/ISH analysis.
