# Supplementary figures and images for: CCN3 is dynamically regulated by treatment and disease state in multiple sclerosis
Source: J Neuroinflammation. 2020 Nov 22;17:349. doi: 10.1186/s12974-020-02025-7 (PMC7681974; doi:10.1186/s12974-020-02025-7)

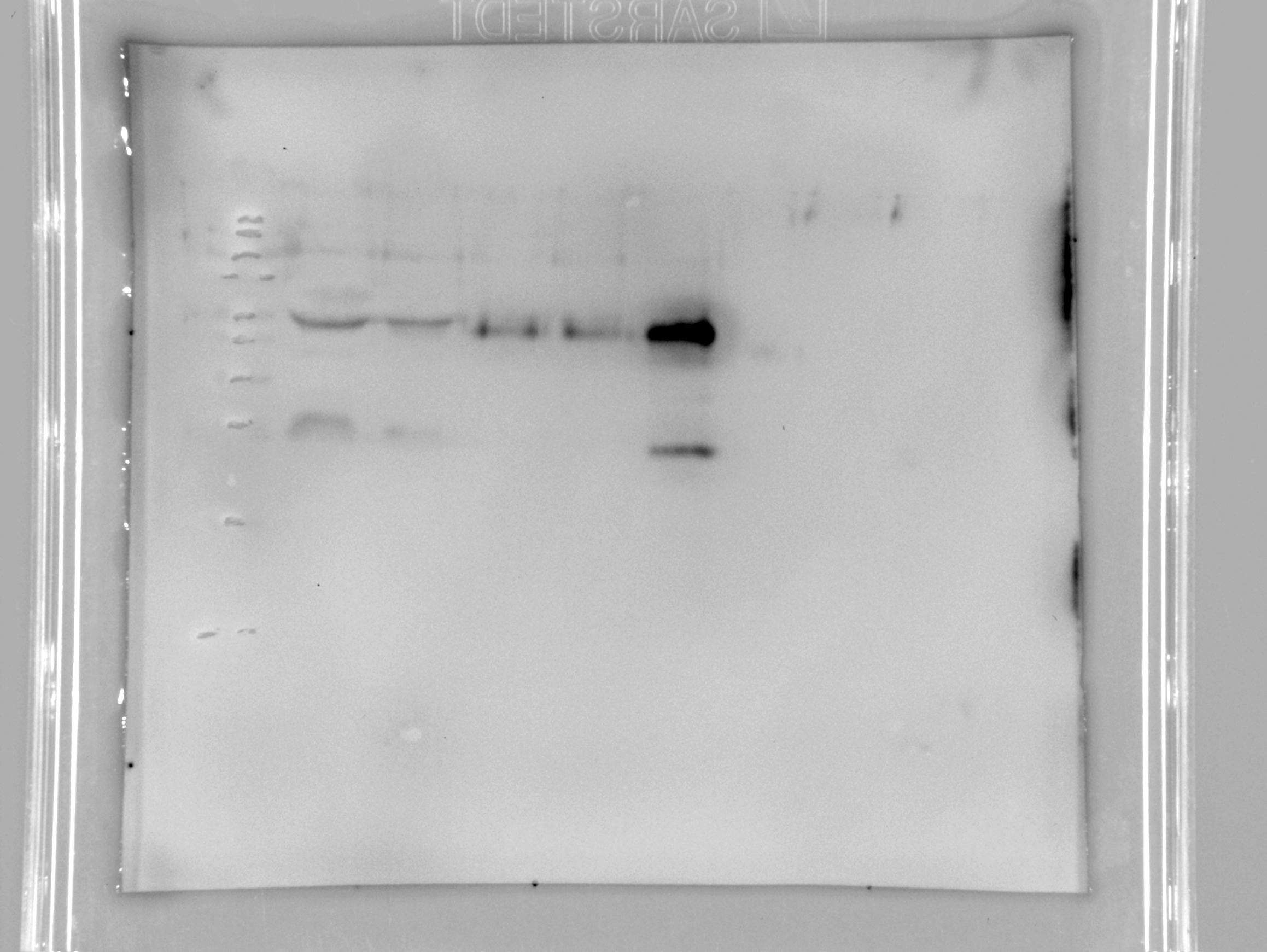

Supplement: Supplementary file 2 — Additional file 2. Fig. 4a raw image of western blot. [file 12974_2020_2025_MOESM2_ESM.tif]
